# Airway Delivery of Encapsulated Cytokine-Secreting Cells for Local Immunomodulation in Inflammatory Lung Diseases

**DOI:** 10.21203/rs.3.rs-8051602/v1

**Published:** 2025-11-27

**Authors:** Omid Veiseh, Samira Aghlara-Fotovat, Kailyn Nunez, Miguel Mendez-Sosa, Michael Guinn, Saad Malik, Saliha Pathan, Jacob Cabler, Maheshwari Ramineni, Suridh Chakravarty, Julia Goldman, Michael Diehl, Ravi Ghanta

**Affiliations:** Rice University; Rice University; Rice University; Baylor College of Medicine; Rice University; Baylor College of Medicine; Baylor College of Baylor; Rice University; Houston Methodist Research Institute; Baylor College of Medicine; Baylor College Of Medicine; Rice University; Baylor College of Medicine

## Abstract

Dysregulated lung immunity drives excessive inflammation, leading to diseases like acute respiratory distress syndrome (ARDS) and pulmonary fibrosis that have high morbidity and mortality. Immunomodulation can attenuate inflammation and improve outcomes, however off-target effects from systemic delivery lead to adverse events that limit clinical translation. Herein, we develop a modular cell-based microcapsule platform that can be administered via the airway for localized, durable, and tunable delivery of various immunomodulatory proteins to the lungs. With this system, we demonstrate therapeutic efficacy of localized delivery of two proteins, interleukin-10 (IL-10) and interleukin-1 receptor antagonist (IL-1Ra), in a lipopolysaccharide-induced rodent model of ARDS. Single-cell RNA sequencing revealed that IL-10-secreting capsules reprogram the lung immune landscape in ARDS by altering myeloid cell composition, suppressing pro-inflammatory gene expression, and promoting the resolution of inflammation. In a bleomycin pulmonary fibrosis model, the platform enables durable and sustained delivery of IL-10 to alleviate hypoxemia and rescue lung architecture. Safety and biocompatibility were further confirmed in a large animal model, highlighting the clinical potential of the platform for the treatment of inflammatory lung diseases.

Immunomodulation is a promising strategy for treating a range of inflammatory diseases of the lung, but successful clinical application requires targeted, localized delivery of therapeutic agents. The lungs serve as a first-line defense against the external environment, constantly exposed to many inhaled pathogens, allergens, and environmental pollutants^1^. As such, the immune response within the respiratory tract is carefully regulated, shifting between heightened pathogen clearance and tissue repair^2^. Dysregulation of these processes, and particularly an excessive unresolved inflammatory response due to pulmonary infections or indirect insults, such as sepsis or trauma, can result in conditions like acute respiratory distress syndrome (ARDS) and pulmonary fibrosis. These disorders are characterized by excessive inflammatory cell infiltration, pro-inflammatory and fibrotic signaling, and dysfunctional tissue remodeling, leading to architectural destruction and compromised gas exchange. Mortality rates of ARDS range between 35%−46%^3–5^, with a subset of survivors developing a fibroproliferative response, placing them at risk of developing pulmonary fibrosis^3, 6–8^. Therapeutic strategies for these conditions remain limited, as current interventions for ARDS provide primarily symptomatic relief and supportive care, and pulmonary fibrosis lacks a cure, limited to two available drugs or a lung transplant^9, 10^.

Advancements in targeting the immune environment of the lung to restore immune homeostasis have largely focused on micro- and nanoparticle-based inhalational delivery of anti-inflammatory corticosteroids, thereby reducing systemic toxicity^11–14^. However, these drugs only broadly suppress rather than precisely modulate immune responses, and their clinical efficacy remains highly variable among patients^15, 16^. To more precisely modulate inflammation, the immune system’s endogenous signaling molecules can be leveraged as therapeutic agents, but requires newer strategies for local delivery. Inhaled mRNA lipid-based nanoparticles are a promising approach for stimulating protein production in the lungs to coordinate immune cell activation, reduce inflammation, and promote repair, but remains limited by short-duration, limited potency, and poor lung penetration^17–21^. Cell based protein delivery may overcome these limitations by altering immune environment through paracrine signaling^22–24^, or as drug carriers^25–27^, and can offer sustained production and delivery of therapeutic proteins directly to targeted tissues^28–30^. Mesenchymal stromal cells (MSCs), for example, have been reported to alleviate severe acute lung injury through their anti-inflammatory paracrine factors secreted in response to the inflammatory environment^31–33^. However, MSCs rely heavily on their microenvironment, making their secretory output variable^34, 35^. Other cell-based approaches remain constrained by significant challenges associated with achieving precise dosing, sustained cell viability, long-term durability, and effective multiplexed protein delivery^29, 36^.

Cytokines play an essential role in immunity, controlling cellular responses to create or reduce inflammation^37^. Their heighted pro-inflammatory signaling cascade contributes to the pathogenesis of acute and chronic inflammatory lung diseases^38, 39^ Directly targeting these signaling molecules has been explored through monoclonal antibodies for mediators of inflammation such as granulocyte–macrophage colony-stimulating factor (GM-CSF)^40, 41^ and interleukin-6^42^. Among the various immunomodulators explored, IL-10 and IL-1Ra have emerged as particularly promising candidates for resolving lung inflammation (NCT05914454, NCT04330638, NCT04680949, NCT04364009)^43–45^. IL-10 is a potent anti-inflammatory cytokine with established roles in limiting immune activation and promoting tissue repair,^46, 47^ while IL-1Ra counteracts IL-1–driven inflammatory cascades that contribute to acute injury and fibrosis^48^. Both have shown efficacy in reducing cytokine production, immune cell infiltration, and tissue damage in preclinical and clinical studies of ARDS and related inflammatory lung disorders. However, their short half-life and broad anti-inflammatory effects highlight the need for localized and sustained delivery platforms to fully realize their therapeutic potential^49^.

To address these issues, we developed a modular platform optimized for localized delivery of cell-generated biologics to the lungs. To build this, we leveraged a cell chassis with previous FDA-approved clinical use^50^. Combined with cell engineering and encapsulation strategies, the platform offers the ability to tune cell-generated protein type and dosing, allowing for efficient delivery of immune regulatory molecules to the lungs. Leveraging rat models of ARDS and pulmonary fibrosis, we assessed the system’s ability to facilitate suppression of inflammation and promote repair, exploring immune cell activation through single-cell RNA sequencing. To evaluate safety and facilitate clinical translation, we examined bronchoscope-mediated delivery in a large animal model, examining biocompatibility and the ability to instill therapy to specific anatomic lung regions, a feature with potential clinical ability to treat specific pathologic regions.

## Results

### Alginate encapsulation enhances cell delivery and retention in the lungs

**Figure 1:**
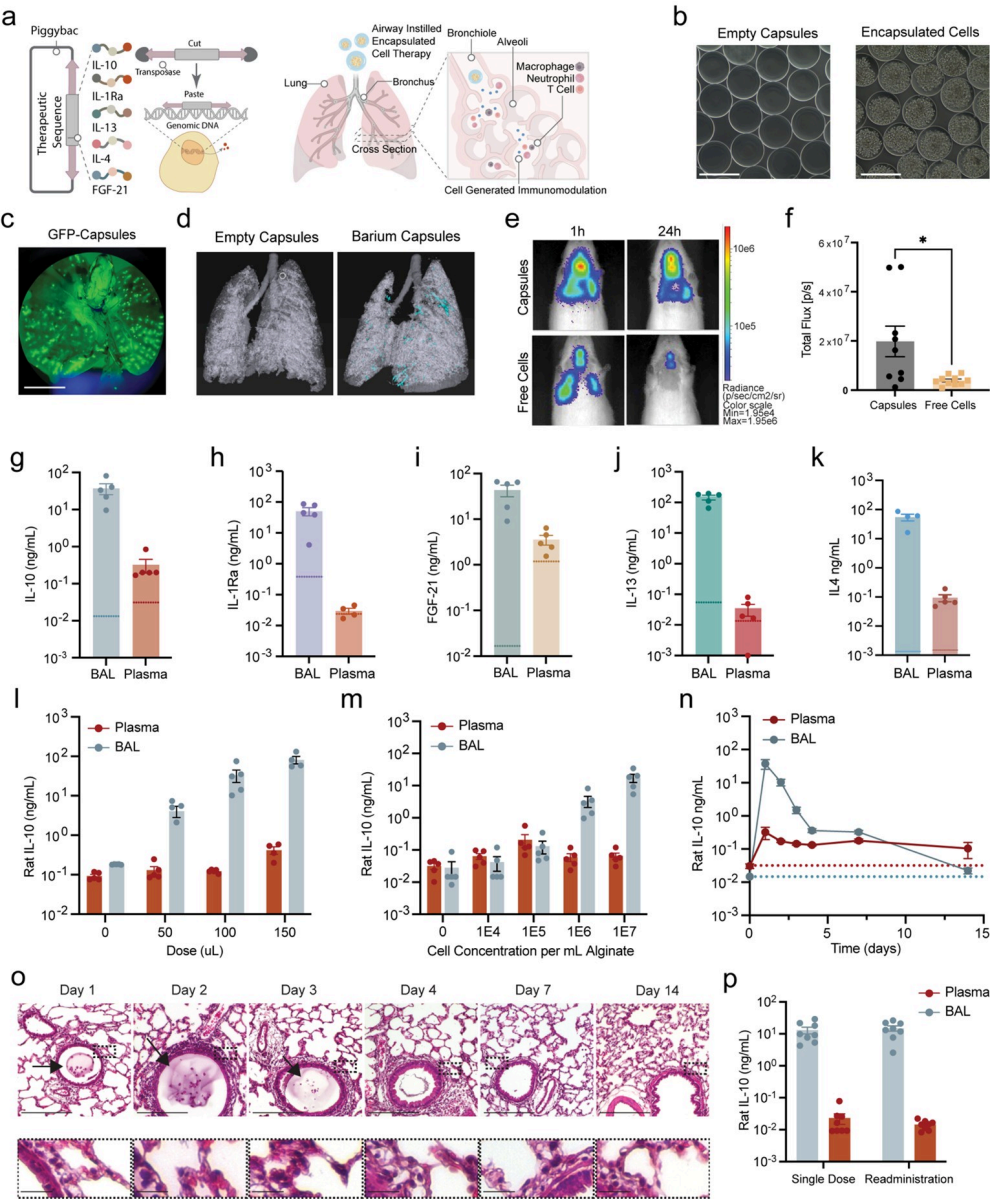
Characterization of a microcapsule delivery platform for localized immunomodulation. **A)**Schematic of piggyBac transposon-mediated engineering technique used to develop a library of therapeutic proteins with schematic of intratracheal instillation of alginate hydrogels loaded with retinal pigment epithelial (RPE) cells. Inset shows interaction between alginate capsules engineered to produce immunomodulatory proteins and local immune cells within bronchioles. **B)**Representative brightfield image of alginate capsules and cell-laden capsules. Scale bar, 400 mm **C)** Ex vivo fluorescent imaging of dextran-loaded capsules distributed throughout the lungs immediately after instillation. Scale bar, 1 mm. **D)** MicroCT scans of rat lungs after instillation of empty or barium sulfate-loaded capsules. **E)** In vivo bioluminescent imaging of firefly luciferase expression cell capsule retention versus an equivalent dose in unencapsulated, free cells. 60s exposure time (n=9–10 rats/group) **F)** In vivo bioluminescent total flux analysis 24-hours post-instillation. Analyzed using an unpaired two-tailed Welch’s t-test. *P=0.0342 (n = 9–10 rats/group) **G-K)** Quantification of bronchoalveolar lavage (BAL) fluid and plasma concentrations of IL-10, IL-1Ra, FGF-21, IL-13, and IL-4 24 hours post-instillation of 100 ml of 1E7 cells/mL capsules. The dashed line represents basal levels. **L-M)** IL-10 dose escalation measured in BAL and plasma 24 hours post-instillation with L) increasing capsule volume (saline, 50, 100, and 150 μL of 1E7 cells/mL RPE-IL-10 capsules) and M) increasing cell density (100 μL of empty capsules, 1E4, 1E5, 1E6, 1E7 cells/mL of alginate) **N)** BAL and plasma concentrations of IL-10 as a function of time with a dose of 100 μL of 1E7 cells/mL RPE-IL-10 capsules. Dashed line represents basal levels. **O)** Representative histology images corresponding to time points in panel M. Scale bar, 200 mm and 25 mm **P)**BAL and plasma concentrations of IL-10 24 hours post-instillation with RPE-IL-10 capsules and upon repeat dose at 30 days. (n=8 rats/group). Unless otherwise specified, all other quantification data are represented as mean ± SEM (n=5 rats/group).

ARPE-19 cells were engineered to express different immunomodulatory cytokines, including IL-10, IL-1Ra, Fibroblast growth factor-21 (FGF-21)^51^, Interleukin-13 (IL-13)^52^, and Interleukin-4 (IL-4)^52^ via piggybac-mediated integration methods^53, 54^ (Fig. 1a). The resultant cytokine secreting cells were subsequently encapsulated within alginate hydrogels using established methods^54–56^. Alginate hydrogels are commonly used as a protective polymer to encapsulate a variety of cargo, such as cells and soluble drugs, allowing for intratracheal delivery into murine lungs^56–58^. To determine the most suitable size for intra-tracheal instillation, we first developed alginate hydrogel microcapsules of varying diameters—300 am, 600 μm, 800 μm, and 900 μm (Fig. 1b, **Supplemental Fig. 1a**). Consistent size distribution was confirmed through brightfield imaging (**Supplemental Fig. 1b**). The 300 μm microcapsules were selected for further studies based on their ability to navigate the small airways of the rat lung, minimizing the risk of airway obstruction^59, 60^. Cells encapsulated within 300 μm microcapsules maintained >90% viability (**Supplemental Fig. 1c-d**). While larger capsules could be advantageous in human applications due to larger airway dimensions, the 300 μm size offered the optimal balance between effective delivery and safety in the rat model.

Delivery and retention of the therapeutic platform within the lung were evaluated via ex vivo and in vivo imaging of rat lungs after intratracheal instillations of the 300 μm microalginate capsules loaded with fluorescent reporting molecule FITC-labeled dextran, barium sulfate-loaded capsules, or genetically engineered RPE cells that constitutively express firefly luciferase. Lungs imaged ex vivo after delivery of FITC-labeled dextran-loaded capsules showed even distribution within the lungs (Fig. 1c). Furthermore, CT scans taken 24 hours after instillation of barium-filled capsules further confirmed successful pulmonary delivery and broad dispersion of the capsules across both lungs (Fig. 1d). Encapsulated firefly luciferase-expressing cells exhibited higher luminescence signals and maintained their localization in the rat lung for up to three days. In contrast, signals from unencapsulated cells decayed more rapidly and persisted for only one day, confirming that the delivery of encapsulated cells can improve cell delivery and retention within the lung compared to unencapsulated free RPE cells (Fig. 1e–f, **Supplemental fig 1e**). Similar uniform delivery and persistent retention were also observed with lung instillation of 300-μm capsules in mice, which were evenly distributed throughout the lungs and remained localized for up to seven days (**Supplemental Fig. 1e-f**).

### Alginate encapsulated cells facilitate local protein delivery with tunable dosing

To examine the modularity of our platform, RPE cells engineered to stably express individual immunomodulatory cytokines (IL-10, IL-1Ra, FGF21, IL-13, or IL-4) were encapsulated within 300 μm alginate microcapsules and tested for cytokine production. In vitro quantification demonstrated capsules consistently produced and released significant amounts of these proteins over a 24-hour period (**Supplemental Fig. 1h-l**). We proceeded to evaluate the localized presence of the microcapsules in vivo by quantifying the concentrations of IL-10, IL-1Ra, FGF-21, IL-13, and IL-4 in bronchoalveolar lavage (BAL) fluid and plasma 24 hours after intratracheal instillation in rats (Fig.1g–k). Pronounced fold-change between local (BAL) and systemic (plasma) concentrations were observed, with local concentrations being at least 10-fold and up to 10,000-fold higher than systemic levels. This finding underscores the platform’s ability to confine the therapeutic effect to the lungs, which is essential to minimizing systemic side effects and highlighting the importance of localized delivery in treating lung-specific inflammatory conditions.

We next conducted dose-response studies by varying the volume of 300 μm microcapsules (saline, 50, 100, and 150 μL) and then quantified and compared local and systemic IL-10 concentrations. IL-10 concentration in BAL fluid increased with capsule dose volume (6.59-fold for 50 vs 100; 2.45-fold for 100 vs 150). In contrast, plasma concentrations remained unchanged, indicating delivery is highly localized to the lung (Fig. 1l). We also examined the effect of varying the cell density within the alginate microcapsules on IL-10 production as an alternative method to dose escalation. 100 ul of capsules generated with 1E4, 1E5, 1E6, and 1E7 cells/mL of alginate were administered, with empty capsules serving as the control (Fig. 1m). This approach yielded similar dose-dependent increases in BAL IL-10 concentration with no significant changes in systemic levels.

### Alginate microcapsules enable persistent IL-10 delivery and reiterative dosing.

IL-10 was selected for follow-up studies due to its well-documented anti-inflammatory and anti-fibrotic properties^61, 62^. IL-10 levels were measured in each lung lobe homogenate 24 hours after instillation to confirm uniform cytokine distribution throughout the lungs, supporting tissue accumulation. Consistent IL-10 levels across the different lobes were observed (**Supplemental Fig. 1m**). We next examined the persistence of exogenous IL-10 by monitoring concentrations in BAL fluid and plasma over time following a single intratracheal instillation of 100 μL of 300 μm RPE-IL-10 capsules. Local IL-10 concentrations in the BAL peaked 24 hours post-instillation and declined gradually over five days. IL-10 levels did not fully return to baseline levels until day 14. These baseline levels at this day notably matched those at day 100 (Fig. 1n, **Supplemental Fig. 1n**). In contrast, intratracheal instillation of free IL10 protein resulted in significantly lower BAL concentrations at 24 hours, with cytokine levels returning to baseline by day 2 (32.7-fold lower, **Supplemental Fig. 1o**). Additionally, pleural implantation of larger, 1.5 mm IL-10 capsules produced minimal IL-10 accumulation in the BAL fluid, highlighting the utility of direct airway delivery for effective local cytokine exposure (**Supplemental Fig. 1p**).

Histological analysis of lung tissue after IL-10 capsule instillation indicated the elimination of the microcapsules, which were no longer detectable by day 7 (Fig.1o). To evaluate the platform’s potential for sequential dosing, we measured IL-10 levels 24 hours after an initial instillation and again 24 hours after a second instillation 30 days later. The results revealed no significant difference in local IL-10 concentrations between the first and second doses, indicating that the microcapsule platform does not elicit an anti-drug antibody response (Fig. 1p). Together, these results suggest the microcapsule platform can provide abilities to sustain IL-10 delivery by bolstering therapy cell retention and by enabling repeated dosing without the risk of diminishing efficacy due to immune interference.

### Localized Cytokine Therapy Demonstrates Efficacy in a LPS Model of ARDS

**Figure 2:**
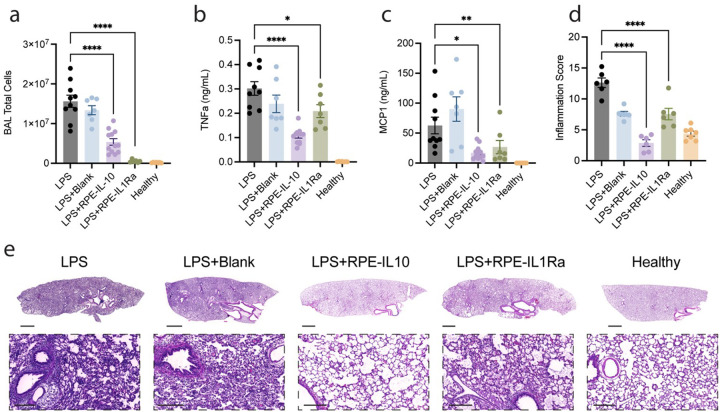
Evaluation of Micro-Capsules in an LPS model of ARDS. **A**) Total number of cells collected from the BAL fluid 24 hours after LPS and capsule treatment determined by Trypan Blue stain. LPS versus RPE-IL10 ****P<0.0001; LPS versus RPE-IL1Ra ****P<0.0001 **B)**The concentration of TNF-α and **C)** MCP-1 in BAL fluid was measured by ELISA from rats receiving treatment as described and harvested 24 hours after LPS administration. TNF-α: LPS versus RPE-IL10 ****P<0.0001; LPS versus RPE-IL1Ra *P=0.0481; MCP1: LPS versus RPE-IL10 *P=0.0191; LPS versus RPE-IL1Ra *P=0.00751; **D**) Quantified histology of the inflammation area at 1 day following LPS administration; LPS versus RPE-IL10 ****P<0.0001; LPS versus RPE-IL1Ra ****P<0.0001 **E)** Scanned images of H&E-stained lungs 24 hours following LPS administration and treatment. Scale bar, 2 mm. Inset is a magnification of a representative section from each lung and depicts the pathology described in the scoring metrics. Scale bar, 200 mm. All data is represented as mean ± SEM (n = 7–12 rats/group), calculated using one-way ANOVA followed by Tukey’s multiple comparison test

To assess the therapeutic efficacy of our cytokine delivery platform in mitigating localized inflammation, we utilized a lipopolysaccharide (LPS)-induced acute respiratory distress syndrome (ARDS) model in rats. ARDS is marked by the rapid onset of severe inflammation and immune cell infiltration, where uncontrolled responses can exacerbate tissue injury. Our goal was to attenuate inflammatory cell recruitment and cytokine production to improve organ health. To this end, rats were euthanized at 24 hours post-LPS instillation (Fig. 2a), and efficacy was evaluated via histological analysis and quantification of local inflammatory cytokine levels

Rats received intratracheal administration of LPS alone or in combination with capsules producing rat IL-10 or IL-1Ra, with LPS, blank capsules, and healthy rats serving as controls (Fig. 2a). Cell counts from the BAL fluid on day one showed a significant reduction in total immune cell counts across all treatment groups compared to untreated LPS controls (IL-10: 2.90x decrease; IL-1Ra: 28.83x decrease). Notably, cell counts in treatment groups were comparable to those in healthy controls, while untreated LPS rats showed a substantial increase (86.89x increase) (Fig. 2a).

To further characterize inflammatory modulation, we quantified the levels of the pro-inflammatory cytokine tumor necrosis factor-alpha (TNFa), and the chemokine Monocyte chemoattractant protein-1 (MCP-1) in the BAL fluid via ELISA. TNFα levels showed significant reductions with IL-10 (2.82x decrease), IL-1Ra (1.45x decrease) compared to untreated controls (Fig. 2b). Treatment groups exhibited MCP-1 levels comparable to healthy rats, with IL-10 treatment achieving the most pronounced reduction relative to LPS controls (3.72x decrease) (Fig. 2c). These findings demonstrate that our cytokine delivery platform rapidly suppresses local immune cell infiltration and reduces pro-inflammatory cytokine levels in an LPS-induced ARDS model. IL-10 and IL-1Ra treatments restored cytokine profiles and cellular counts to near-physiological levels, underscoring their potential to resolve inflammation in a controlled, localized manner.

To investigate the downstream organ-level effects of rapid local immunomodulation, lung tissue was histologically assessed using H&E staining (Fig. 2d–e). The right lung from each rat was harvested and stained to evaluate the extent and persistence of intrabronchiolar inflammation. Quantification of the histological sections based on inflammatory area revealed significantly greater inflammation in the disease group compared to healthy controls (Fig. 2d). Lungs exposed to LPS with blank capsules (1.66-fold) and lungs exposed to LPS with IL-1Ra capsules (1.67-fold) had a reduction in inflammatory areas, approaching healthy levels. Lastly, lungs exposed to LPS with IL-10 treatment elicited a significant reduction in inflammation (4.42-fold), with inflammation scores statistically indistinguishable from healthy controls. The observation in functional improvement with IL-10 led us to proceed with single-cell RNA sequencing of lungs exposed to LPS with IL-10 treatment.

### Single Cell Sequencing Reveals a Predominantly Myeloid-Derived Population:

**Figure 3:**
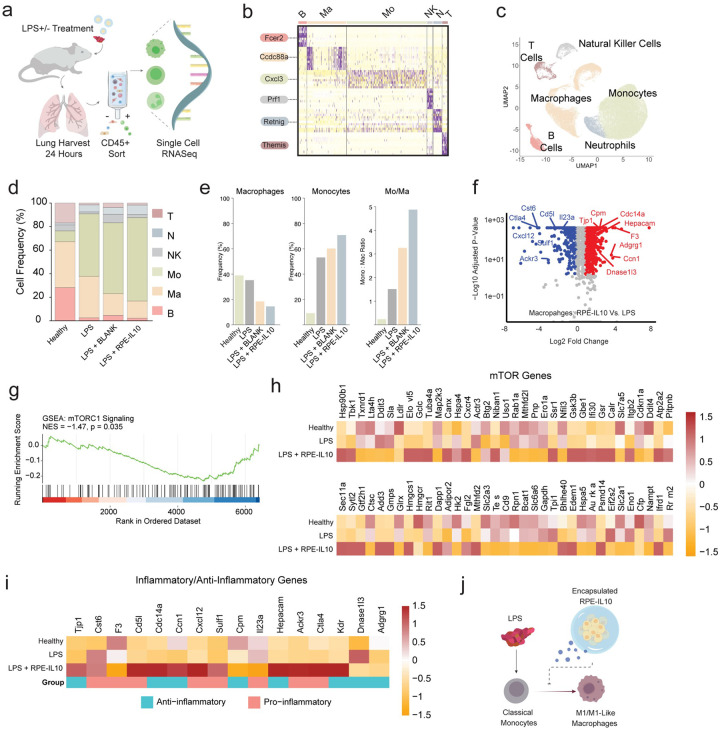
Single Cell Sequencing Identifying Cell types in Healthy, Damaged, and Therapy-Treated Lungs. **A)** Schematic illustration of experimental setup. **B)** Heatmap of highest expressed genes used to label cell clusters. ‘Mo’ - monocytes, ‘Ma’ - macrophages, ‘B’ - B cells, ‘NK’ - natural killer cells, ‘N’ - neutrophils, ‘T’ - T cells. Each row is a different gene with purple color being more highly expressed and yellow less expressed. **C)** UMAP of integrated single cell RNA sequencing data showing distribution of 8 cell clusters. Each dot is a single cell. **D)** Frequency distribution graph showing the clusters in panel C. Each color is a different cell type. **E)**Bar graphs of macrophages, monocytes, and ratio of monocytes to macrophages. **F)** Volcano plots as a comparison of IL-10-treated rats vs LPS exposed rats of macrophages. Red dots are upregulated genes, blue dots are downregulated genes, and gray dots are genes that are not statistically different between conditions. Red genes labeled are significant anti-inflammatory genes and blue labeled genes are significant inflammatory genes. **G)** Gene set enrichment analysis for differential genes between IL-10 treated group and LPS control for mTOR pathway **H)** Heatmap of mTOR genes found significant in GSEA in panel **G. I)** Heatmap of inflammatory and anti-inflammatory genes found significant from volcano plot in panel **F. J)** Schematic illustration of proposed mechanism of IL-10 on monocytes and macrophages.

Integration of molecular and histological analyses across the treatment groups strongly suggested that IL-10 exerts both rapid and sustained immunomodulatory effects in mitigating ARDS-associated inflammation. To elucidate the cellular mechanisms by which IL-10 was creating this observed phenomenon, we performed single-cell RNA sequencing (scRNAseq) of CD45+ cells sorted from healthy lungs, lungs exposed to LPS alone or in combination with either blank or IL-10-producing capsules (Fig. 3a). Analysis of these experimental conditions revealed a predominantly myeloid-derived population along with a small B cell and T cell population (**Supplementary Fig. 2**).

To better characterize these myeloid, T cell, and B cell clusters, we performed cluster analysis of the integrated scRNAseq data set for both highly expressed and canonical markers, revealing six different cell clusters (Fig. 3b & **Supplementary Fig. 3**). Clusters were delineated using a combination of the highly expressed genes in Fig. 3b, the broad cell category identification (**Supplementary Fig. 2**), and canonical markers, revealing the six clusters as monocytes, macrophages, B cells, natural killer cells, neutrophils, and T cells (Fig. 3c). Quantification of the cell numbers across experimental conditions indicated the IL-10 treated group had the lowest macrophage cell signature and highest monocyte population compared to the disease model (Fig. 3d). Subsequent analysis of cell frequency confirmed that the monocyte-to-macrophage ratio was maximally increased following IL-10 administration (Fig. 3e).

Given that IL-10 had the strongest effects in changing macrophage numbers, we turned to analyzing gene expression patterns in this population. The volcano plot comparing the IL-10 capsule group to the LPS-only control indicated a shift towards an anti-inflammatory state, with numerous upregulated anti-inflammatory genes and downregulated inflammatory genes (Fig. 3f). Additionally, gene set enrichment analysis (GSEA) showed that, across all cell types, the mTOR pathway was significantly downregulated in the IL-10 treatment group relative to the disease group (Fig 3g, Normalized Enrichment Score (NES) = −1.47, p=0.035). Importantly, there was a distinct increase in expression of DNA Damage-Inducible Transcript 4 (*Ddit4*), an inhibitor of the mTOR pathway, as compared to healthy and LPS treated rats^63^.

Based on this GSEA finding, we moved to investigate the individual genes in the mTOR pathway which showed clear differences in the healthy, LPS only, and IL-10 treatment groups (Fig. 3i). For example, many genes that were upregulated in the LPS disease model compared to healthy lungs (*Eif2s2, Tpi1, Sla*) were downregulated towards the healthy line state in lung treated with IL-10-producing capsules. Additionally, many genes that were downregulated in the LPS disease model compared to healthy lungs (*Ifrd1, Gsk3b, Edem1*) were upregulated towards the healthy line state in IL-10-producing RPE capsules treatment (Fig. 3h).

Lastly, to investigate IL-10 effects on other inflammatory genes outside of the mTOR pathway, we also investigated a set of inflammatory and anti-inflammatory genes across all cell types (Fig. 3i). Consistent with the mTOR pathway results, we found that treatment with IL-10 increased specific anti-inflammatory genes (*Adgrg1, Cpm, Ccn1*) and down-regulated several pro-inflammatory genes (*Cst6, Ctla4, Tjp1*) relative to the lungs exposed to LPS only. Collectively, these results pointed towards the IL-10 playing an active role in inhibiting monocyte differentiation into inflammatory monocytes or macrophages within the lung tissue (Fig. 3j).

#### Microcapsule Delivery of IL-10 Restores Pulmonary Architecture in a Bleomycin-Induced Model of Pulmonary Fibrosis

**Figure 4:**
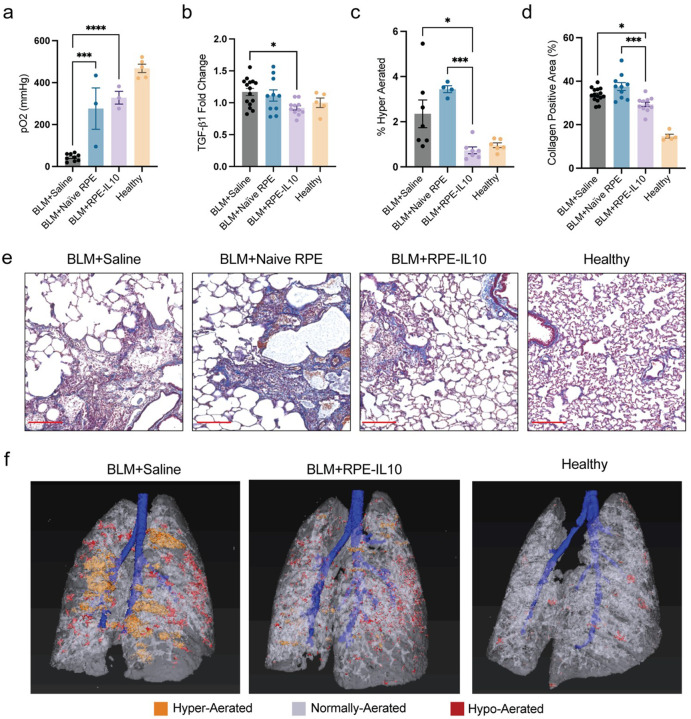
Evaluation of Micro-capsule mediated IL-10 delivery on a rat model of bleomycin induced lung fibrosis **A)** Arterial blood gas measurement of partial pressure of oxygen (pO2); Saline versus Naïve-RPE ***P=0.0005; Saline versus RPE-IL10 ****P<0.0001 **B)** TGF-b1 gene expression in lung tissue. Saline versus RPE-IL10 *P=0.0224 **C)** Quantification of hyper-aerated areas measure by CT imaging. Saline versus RPE-IL10 *P=0.0189; Naïve-RPE versus RPE-IL10 ***P=0.0008 **D)**Quantification of trichrome staining shown as percent collagen-positive area relative to total lung area; Saline versus RPE-IL10 *P=0.0388; Naïve-RPE versus RPE-IL10 ***P=0.0002 **E)** Masson’s Trichrome-stained lung sections. **F)** Representative CT images highlighting aeration levels. All data is represented as mean ± SEM (n = 7–12 rats/group), calculated using one-way ANOVA followed by Tukey’s multiple comparison test.

To evaluate long-term therapeutic efficacy of the platform, we used a repeated-dose bleomycin (BLM) model of pulmonary fibrosis in rats. Animals received five oropharyngeal BLM challenges (days 0, 1, 2, 5, and 6), followed by delivery of IL-10–secreting RPE capsules, naïve-RPE capsules, or saline on day 7. Pulmonary function assessed on day 21 indicated severe hypoxemia in the saline control group, with a 10-fold reduction in the arterial partial pressure of oxygen (pO2) compared to healthy, measured with arterial blood gas (ABG) results taken from rats ventilated with 100% supplemental oxygen. Treatment with IL-10 capsules yielded the greatest improvement, with a 7.4-fold increase over saline-treated animals, approaching healthy values (Fig. 4a).

Lungs treated with IL-10 capsules additionally exhibited a reduction in TGF-b1 gene expression, a master regulator of fibrosis^64^, compared to the saline control (Fig. 4b). CT and histological evaluation further showed IL10 capsule treatment restored pulmonary architecture to near-healthy levels. The volume of hyper-aerated tissue, measured by density of Hounsfield units, in IL-10-treated animals was significantly lower than those treated with saline or naïve-RPE, confirming reduced air trapping and destructive emphysematous changes, demonstrating a protective effect from alveolar destruction (Fig. 4c). Histological assessment using trichrome staining confirmed a reduction in collagen-positive lung area following IL10 treatment. Fibrosis was significantly decreased relative to saline and naïve-RPE groups. No difference was observed between saline and naïve-RPE groups (Fig. 4d–e). CT imaging correlated to the histology staining, showing an increase in parenchyma lesions, including increased hyper-aerated regions the saline control group. IL-10 capsule treatment was found to reduce the development of these lesions across the whole lung (Fig 4f). Together, these findings demonstrate that IL-10–secreting RPE capsules markedly improve lung function, suppress fibrotic remodeling, and restore pulmonary architecture in a chronic model of lung fibrosis.

### Microcapsule Delivery Demonstrates a Favorable Safety Profile for Localized Immunomodulation in Healthy Yucatan Pigs

**Figure 5:**
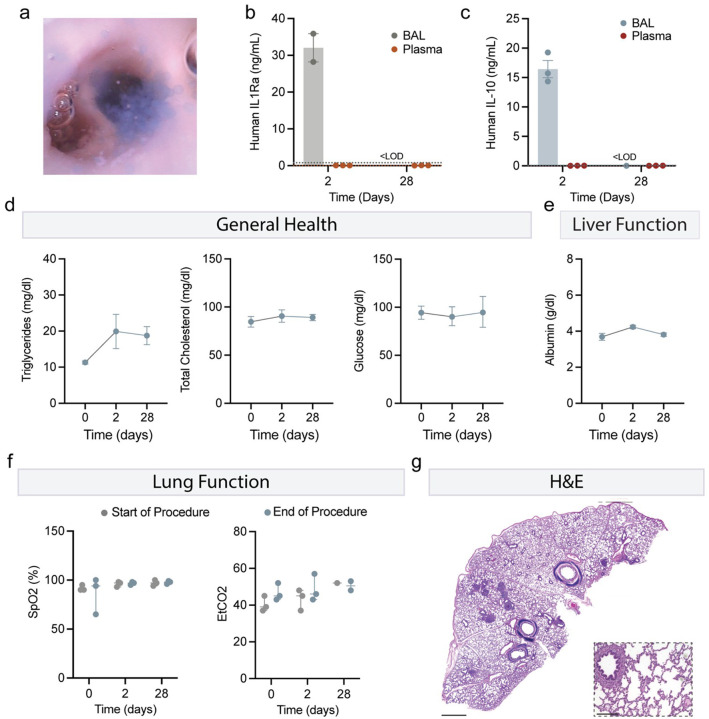
Evaluating the safety profile of microcapsule delivery for localized immunomodulation in healthy Yucatan Pigs. **A**) Demonstration of capsule delivery in pig cadaver lungs. Capsules were dyed blue for visualization **B-C**) Quantification of bronchoalveolar lavage (BAL) fluid and plasma concentrations of IL-1Ra and IL-10 before instillation, two days after, and at euthanasia on day 28 **D**) Triglycerides, Total Cholesterol, and Glucose blood levels on days 0, 2, and 28, showing no significant change. **E**) Albumin blood levels as a marker of liver function showing no significant change. **F**) Saturation of peripheral oxygen and end-tidal carbon dioxide at the start and end of each procedure **G)** H&E-stained sections of the lung. Scale bar, 2 mm and inset, 200 mm. Data represented as mean ± SEM (n=3).

A significant proportion of ARDS patients require mechanical ventilation, with up to 75% of those with severe ARDS depending on this intervention.^4, 5^ Mechanical ventilation presents a viable therapeutic delivery strategy, offering direct access to the inflamed lung via intubation. To evaluate this approach, we encapsulated human IL-10 and human IL-1Ra-expressing RPE cells within our hydrogel platform. Using a bronchoscope, we delivered 5 mL of IL-1Ra capsules and 4 mL of IL-10 capsules directly into the right lower lobe lungs of Yucatan pigs (Fig. 5a).

IL-1Ra and IL-10 concentrations in the BAL fluid were significantly elevated on day 2, observing a 40-fold and 300-fold increase, respectively, from baseline levels measured pre-instillation (Fig. 5b–c). Systemic levels of IL-10 and IL-1Ra remained unchanged compared to pre-delivery levels, confirming localized cytokine release. By day 28, BAL cytokine levels returned to baseline, indicative of a natural clearance mechanism within the lung environment. These results demonstrate the feasibility of delivering cytokine-secreting cell therapies via mechanical ventilation, achieving potent localized immunomodulation while minimizing systemic exposure.

To further assess the systemic effects of intrabronchiolar delivery of hydrogel microcapsules, we evaluated several metabolic markers associated with general health, including triglycerides, total cholesterol, and glucose. Across all three markers, there were no statistically significant differences between baseline (pre-capsule) values and those observed at days 2 or 28 post-administration, indicating no disruption to systemic metabolic health (Fig. 5d). To evaluate liver function, we measured serum albumin levels, a key marker of hepatic protein synthesis and overall liver health. Consistent with the metabolic markers, serum albumin levels showed no significant changes at either day 2 or day 28 compared to baseline values prior to capsule administration (Fig. 5e). These findings collectively suggest that hydrogel microcapsule delivery does not adversely affect systemic metabolic health or liver function over the evaluated time frame.

At each intubation point throughout the study, measurements of peripheral oxygen saturation (SpO_2_) and end-tidal carbon dioxide (EtCO_2_) were recorded to monitor respiratory function and assess potential physiological impacts of capsule delivery. At all-time points, there were no significant changes or unresolved abnormalities in these parameters, indicating that the hydrogel microcapsules did not interfere with normal lung function (Fig. 5f).

At the terminal endpoint, bronchoscopy revealed no visual signs of severe inflammation, tissue damage, or distress in lung tissue adjacent to the capsule delivery site (**Supplementary Video 1**). Biopsies were collected from the lung, heart, kidney, liver, and spleen to further evaluate systemic effects. Qualitative assessment of tissue morphology showed no observable signs of toxicity, inflammation, or adverse effects across these major organs (**Supplemental Fig. 4**). Notably, the lung tissue adjacent to the delivery site appeared normal, with no evidence of inflammation, fibrosis, or other pathological changes, underscoring the biocompatibility and tolerability of the treatment.

## Discussion

Effective resolution of inflammatory lung diseases requires local immunomodulation that current systemic or transient delivery methods cannot sustain. Here, we established a biocompatible encapsulated cell-based platform that enables durable, tunable cytokine delivery to the lungs while minimizing systemic exposure. This modular system supports diverse cytokine payloads and achieves high local concentrations to resolve acute inflammation and promote long-term tissue repair. In models of ARDS, IL-10 and IL-1Ra delivery attenuated LPS-induced inflammatory cytokines and reprogrammed the immune landscape toward a reparative phenotype, while in pulmonary fibrosis, the same approach enhanced fibrotic resolution. Single-cell RNA sequencing revealed shifts in myeloid populations, including a reduction in macrophages, consistent with immune reprogramming. Mechanistically, IL-10 suppressed activation of the mTOR pathway, a key regulator of macrophage polarization, providing insight into its anti-inflammatory efficacy across both acute and chronic contexts^65, 66^. Together, these findings establish a versatile and re-dosable platform for precise, localized immune modulation in the treatment of chronic and recurrent inflammatory lung diseases.

Prior efforts at immunomodulation in inflammatory lung disease have evaluated several delivery strategies in preclinical and clinical settings. Local and systemic administration of mesenchymal stromal cells (MSCs) has been pursued for their ability to sense inflammatory cues and secrete immunoregulatory factors^67^, but even with genetic programming or priming, the magnitude, timing, and duration of secretion remain difficult to control in vivo. The efficacy of MSCs is highly dependent on the inflammatory state of the lung^34^, and immune recognition of transplanted cells reduces persistence and consistency of response. Improved retention has been explored through biomaterial encapsulation of MSCs, which enhances local persistence in the lungs and reduces macrophage-mediated clearance^23^, yet this approach still does not address the inherent limitations of MSC-based therapy. mRNA-based therapies offer more direct expression control but remain limited by inefficient delivery, with only about 20% of intravenously administered mRNA reaching the lungs^68^, delayed translation onset of roughly 4–6 hours in even instances of local delivery, and rapid degradation, requiring repeated dosing to maintain effect^18^. Our encapsulated cell-based system addresses these limitations through sustained, environment-independent protein release that achieves rapid therapeutic levels, prolonged local retention, and tunable cytokine output without dependence on host cell uptake or viability

Beyond ARDS and pulmonary fibrosis, this platform offers a range of therapeutic modalities, not just limited to inflammation suppression, broadening the application of cytokines in lung diseases. Proteins that control the innate or adaptive immune response can also be produced by our platform and assist in bacterial clearance in respiratory infections such as bacterial pneumonia^69^. Further, our ability to precisely control local dose of therapeutic proteins enables the delivery of proinflammatory cytokines such as interleukin-2 or interleukin-12, which may otherwise cause severe systemic toxicities, for various lung cancer indications^70, 71^.

Despite promising pre-clinical results, the challenge of disease heterogeneity among patients often hinders clinical translation^72^. Although our current findings are restricted to monotherapy, the inherent flexibility of the platform supports multiplexed cytokine delivery to offer a personalized therapeutic cocktail. The system’s capacity for sequential redosing facilitates dynamic tailoring to fluctuating patient needs. Future refinements could integrate sense-and-response mechanisms into encapsulated cells to enable the timed release of therapeutic proteins in response to specific inflammatory cues, further refining treatment strategies^28, 29^.

In summary, we show a modular, plug and play platform for delivery of cell-generated biologics directly to the airway that is safe and effective in acute and chronic lung injuries. The use of a bronchoscope as the delivery mechanism supports rapid translation into human clinical trials for severe ARDS and pulmonary fibrosis, conditions currently facing high mortality rates and limited treatment options. This versatile system accelerates the testing of novel cytokine therapeutics without the need for extensive protein engineering. The platform can be leveraged not only to create personalized combination therapies but also to explore monotherapies in other lung pathologies. With the ability to ensure rapid therapeutic dosing, extended local presence, and a controllable cytokine output, this approach can easily be extended to diverse lung pathologies, such as ventilator-induced lung injury, cancer, and bacterial infections. Overall, these findings establish a robust microcapsule delivery system as a promising alternative therapeutic platform to current systemic and inhalable approaches.

## Methods

### Cell Engineering

Human Retinal pigment epithelial (ARPE-19) cells were purchased from American Type Culture Collection (ATCC) and have been authenticated by the vendor. All cell lines have tested negative for mycoplasma contamination. Vector Builder was used to design and purchase expression vectors for Rat IL-10 (NM_012854.2), Rat IL-1Ra (NM_022194.2), Rat IL-4 (NM_201270.1), Rat IL-13 (NM_053828.1), Rat FGF21 (NM_130752.2), Human IL-10 (NM_000572.2), Human IL-1Ra (NM_173841.3), and humanized firefly luciferase (Luc2) using the piggyBac backbone, with genes of interest under the CAG promotor with a puromycin resistance gene (pPB[Exp]-Puro-CAG>GOI). The pRP[Exp]-CAG>PBase from Vector Builder was used as the helper plasmid. ARPE-19 cells were transfected using the piggyBac transposon/transposase system via lipofection 3000 (Invitrogen) with a 2:1 ratio of expression vector to helper plasmid following the manufacturers protocol. After one week of selection with puromycin, transfected cells were expanded in DMEM/F12 medium supplemented with 10% fetal bovine serum (FBS) and 1% Antibiotic-Antimycotic. Cells were passaged using 0.25% trypsin.

### Microcapsule Fabrication

Microcapsules were generated via electrostatic spraying as previously described.^55^ SLG20 alginate (1.4% w/v) was dissolved in 0.8% saline and stirred overnight. Before encapsulation, cells were washed three times with calcium-free KREBS buffer and resuspended in alginate at the desired concentration. Capsules were formed by electrostatic spraying of alginate through a syringe equipped with a needle and positioned above a barium chloride crosslinking bath. A syringe pump controlled the infusion rate, while a voltage of 3.0–3.5 kV regulated capsule size, yielding ~300 μm capsules. Capsules were incubated in the crosslinking solution for 15 min, collected using a 70-μm cell strainer, and washed three times in HEPES buffer before transfer to complete media for subsequent use. For the incorporation of fluorescent dextran in the capsules, FITC Dextran (Sigma Aldrich, FD2000S, 2e6kDa) was dissolved at 6mg in saline. 10ul was added per 1 mL of alginate prior to generation of capsules. For the incorporation of barium sulfate for CT scanning, 200 mg of barium sulfate (Sigma Aldrich, B8675) was mixed in 1 ml of alginate prior to generation of capsules.

### Validation of Cytokine Production

Encapsulated cells were aliquoted into 100 μL doses and incubated in 1 mL of media at 37°C for 24 h. Cytokine production was quantified using ELISAs (Abcam), while cell viability within capsules was assessed via live-dead staining (2 μM Calcein AM, 4 μM Ethidium Homodimer in PBS). Capsules were digested with alginate lyase (100 μL EDTA, 5 mg alginate lyase, 5 mL complete media), and cells were enumerated using a Countess automated cell counter.

### Animal Studies

All animal experiments were approved by Rice University’s and Baylor College of Medicine’s Institution Animal Care and Use Committees (IACUC). Prior to administration, capsules were washed three times in sterile saline. Capsules were suspended in saline and delivered intratracheally into Sprague Dawley rats (250–300 g) under 3.5% isoflurane anesthesia. Dose-escalation studies varied capsule volumes (50–200 μL) while maintaining a cell concentration of 10^6^ cells/mL alginate. For cell concentration escalation, capsule volume was held constant at 100 μL, and cell density ranged from 10^4^ to 10^7^ cells/mL alginate.

### In Vivo tracking of capsule distribution

To evaluate capsule distribution in the lungs of rats, luciferase-expressing RPE cells were encapsulated in 300-μm alginate capsules at a density of 10^6^ cells/mL. Rats were anesthetized with 3.5% isoflurane and intubated. One cohort received 100 μL of capsules suspended in 200 μL of saline, while a second cohort received an equivalent dose of unencapsulated cells suspended in 300 μL of saline. Rats were administered IVISbrite D-Luciferin Potassium Salt Bioluminescent Substrate (Revvity) was injected intraperitoneally at a dose of 150 mg/kg body weight, and luminescence was measured one-hour post-injection and daily for three days, using the In Vivo Imaging System (IVIS). For capsule distribution studies in mice, 50 μL of capsules resuspended in 100 μL of saline was administered.

### LPS model of ARDS

To induce acute respiratory distress, rats were anesthetized and via isoflurane 3.5% and placed in a supine position intubated using a 20-gauge catheter. Once intubated, the catheter is connected to a volume-cycled Rat Ventilator Minivent (Harvard Apparatus) and run on supplemental 100% oxygen with a tidal volume of 2.5mL and a respiratory rate of 75 respirations per minute. Following intubation, sterile bland ophthalmic ointment was used on the eyes to avoid corneal drying. Lipopolysaccharide (LPS; L2880) was reconstituted with saline at a concentration of 5 mg/mL and administered directly into the lungs at a dose of 5 mg/kg. For groups receiving capsules, 100 μL of capsules was co-administered with the appropriate volume of LPS. To control for the added volume, untreated control animals received an additional 100 μL of saline alongside the LPS dose.

### Bleomycin model of pulmonary fibrosis

Rats were anesthetized using 3.5% isoflurane and placed in a supine position. Bleomycin (MWI Animal Health) was dissolved in saline and administered on days 0, 1, 2, 3, 5, and 6 through oropharyngeal administration at a dose of 1.66U/kg. Treatment was delivered intratracheally on day 7, and rats were euthanized on day 21. For capsule groups, 50ul of capsules in 150 μL of saline were administered. For saline treatment, 200 μL of saline was administered.

### Euthanasia and Sample collection

At designated time points post-instillation, rats were anesthetized and intubated for bronchoalveolar lavage fluid (BAL) collection. Ice-cold PBS containing 100 μM EDTA (1 mL) was instilled into the lungs and immediately recovered; this process was repeated three times to achieve a total lavage volume of 3 mL. BAL fluid was filtered through a 70-μm cell strainer and centrifuged at 500 × g for 7.5 minutes at 4°C. The resulting supernatant was aliquoted and stored at −20°C for subsequent ELISA analysis.

Blood samples were collected from the inferior vena cava into EDTA-coated tubes. Samples were centrifuged at 2,500 rpm for 10 minutes to separate plasma, which was stored at −20°C until analysis. For arterial blood gas measurements, blood samples were taken from the left ventricle into lithium heparin-coated tubes and measured immediately following collection. The IDEXX VetStat Electrolyte Blood Gas Analyzer was used with the Respiratory/Blood Gases cassette.

### Micro-Computed Tomography

After anesthesia induction with 3.5% isoflurane and maintenance with 2% isoflurane, images were taken in free-breathing rats on day 21 using the Mediso nanoScan^®^ SPECT/CT/PET with the x-ray current of 50kVs and 314 mAs exposure. Respiratory gating was used to create reconstructions at end-expiration phases. Images were analyzed using VivoQuant. The lungs were selected using the otsu method^73^. A region growing segmentation was used to select the trachea and major airway. A semi-automatic thresholding method was used to segment the lungs based on aeration levels^74^.

### Histological Analysis

For histological analysis, the lungs and heart were excised en bloc. The lungs were flushed with 3–5 mL of formalin via direct injection through the right ventricle to preserve tissue architecture. The lungs were then fixed in formalin for 24 hours and then transferred to 70% ethanol. The lower right and left lobes were embedded in paraffin and cut into 5mm sections and stained with hematoxylin and eosin (H&E) and Masson Trichrome. Slides were scanned at 20x and analyzed using Zeiss Zen Lite.

To characterize inflammation of the tissue collected for the various experimental conditions, we developed a custom R code that quantified inflammatory tissue area based on grayscale images. H&E-stained images were converted to grayscale images, had an intensity threshold applied to identify cellular nuclei and inflammatory areas. Inflammatory areas were defined as lower intensity regions on the images. Mean inflammatory areas and standard errors were calculated for each condition. Percent positive collagen was quantified from whole-slide scans of lung tissue stained with Masson’s Trichrome using ImageJ, calculating the percent area of collagen relative to total tissue.

### Single Cell RNA Sequencing (scRNAseq)

Rat lungs were excised from healthy rats and 24 hours after administration of LPS with either saline, empty capsules, or IL-10 secreting capsules. Five lungs per group were collected and combined into final samples. The lung tissue was manually minced and converted to single cells using a multi tissue dissociation kit (Miltenyi Biotec; 130-110-203) according to the manufactures protocol for dissociation of rat lungs. Red blood cell lysis buffer was used to remove erythrocytes following the tissue dissociation. Afterwards, cells were washed with phosphate buffered saline (PBS) and stained with Alexa Fluor^®^ 488 Anti-CD45 antibody [clone MRC OX-1] (Abcam; ab256254) for 1 hour. Cells were washed with PBS and resuspended in FACS buffer. Cells were then stained with NucBlue^™^ (Invitrogen; R37606) to assess for viability and ran on the Aurora CS Cell Sorter to collect 500,000 viable cells. 20,000 cells were then used on a Chromium instrument by 10x Genomics X with 20,000 reads per cell, totaling 400M reads.

### Bioinformatics of scRNAseq Data

The rat (Rattus norvegicus) FASTA information was downloaded and used to create a reference file via CellRanger on a Google Compute Engine VM. Once the reference file was created, it was subsequently uploaded into the 10x Genomics analysis platform. Simultaneously, the FastQ raw data files from sequencing were uploaded and combined (R1-R2-I1-I2) for each sample into the 10x Genomics cloud analysis platform. The sample files were then mapped with the reference and downloaded for further analysis using Seurat in R studio. Data then underwent normalization, removal of cells with outlier findings (e.g. based on RNA features or mitochondrial content), scaled, and visualized with principal component analysis. Data from each condition was integrated into a final Seurat object allowing analysis of the four conditions: healthy lungs, lung exposed to LPS, and lungs exposed to LPS and IL-10-producing capsules. Cell clusters were annotated based on canonical markers found in literature for rats. Cluster and gene expression analysis was performed in R studio like previously done^75^ and by using Seurat software packages^76^. Trajectory analysis was performed using Monocle3^77–79^.

### Quantitative real-time PCR

Total RNA was extracted and purified from flash frozen tissue from the lower left lobe using the Qiagen RNeasy Mini kit. cDNA was synthesised using 1 mg of total RNA using the iScript Reverse Transcription Supermix for RT-qPCR (Bio-Rad; Cat:1708840). qPCR was performed using the iTaq Universal SYBR^®^ Green Supermix (Biorad; Cat: 1725124) on the Bio-Rad CFX96. Relative gene expression was analyzed using the delta delta Ct method with *b-actin* used as the housekeeping gene. Fold change from healthy levels were used.

### Bronchoscope delivery of microcapsules in Yucatan Pigs

Prior to encapsulation, cells were tested for mycoplasma using a commercially available kit (R&D Systems, Inc.). 4mL of Human IL-10 capsules and 5 mL of Human IL-1Ra capsules were made at 10e6 cells/ml of alginate. On day 0, pigs (n=3) were sedated with 4.4mg/kg telazol and 2.2 mg/kg xylazine IM. 5mg/kg of ketamine was given IM if additional sedation was needed. 2–3% Isoflurane was used for maintenance of anesthesia. The pigs were intubated, and the lungs were lavaged using sterile PBS using an endoscope. The capsules were delivered in saline directly to the lungs. On days 2 and 28, the pigs were re-anesthetized and re-intubated, and a lavage of the lungs was performed with PBS. Pigs were euthanized with euthasol on day 28 following the lavage. Biopsies from the lungs, heart, liver, kidney, and spleen were collected for histology. The BALF was centrifuged at 500g for 7.5 minutes at 4°C. The supernatant was frozen at −20°C for subsequent ELISA analysis.

### Enzyme linked immunosorbent assay

Prior to instillation of protein-producing capsules, capsule doses were incubated in 1 mL of media and ran on their respective ELISA kit. Therapeutic and inflammatory protein levels were measured using the BAL fluid. Rat IL-10 (ab214566), Rat IL-1Ra (ab282875), Rat FGF21 (ab223589), Rat IL-13 (ab269547), Rat IL-4 (ab100770), Rat TNFa (ab236712), Rat MCP1 (ab219045), Human IL-10 (ab185986), and Human IL-1Ra (ab211650) ELISAs were purchased from Abcam.

## Supplementary Material

Supplementary Files

This is a list of supplementary files associated with this preprint. Click to download.

- NatMaterialsLungSupplementalFigures.pdf

## Data Availability

All data needed to evaluate the conclusions in the paper are present in the paper and/or the Supplementary Materials. The datasets generated during and/or analyzed during the current study are available from the corresponding author on reasonable request
